# Inflammation and NF-κB Signaling in Prostate Cancer: Mechanisms and Clinical Implications

**DOI:** 10.3390/cells7090122

**Published:** 2018-08-29

**Authors:** Jens Staal, Rudi Beyaert

**Affiliations:** 1 VIB-UGent Center for Inflammation Research, Unit of Molecular Signal Transduction in Inflammation, VIB, 9052 Ghent, Belgium; 2 Department of Biomedical Molecular Biology, Ghent University, 9000 Ghent, Belgium

**Keywords:** prostate, cancer, androgen, castration, inflammation, NF-κB, cytokines, protein kinase C, signaling, clinical

## Abstract

Prostate cancer is a highly prevalent form of cancer that is usually slow-developing and benign. Due to its high prevalence, it is, however, still the second most common cause of death by cancer in men in the West. The higher prevalence of prostate cancer in the West might be due to elevated inflammation from metabolic syndrome or associated comorbidities. NF-κB activation and many other signals associated with inflammation are known to contribute to prostate cancer malignancy. Inflammatory signals have also been associated with the development of castration resistance and resistance against other androgen depletion strategies, which is a major therapeutic challenge. Here, we review the role of inflammation and its link with androgen signaling in prostate cancer. We further describe the role of NF-κB in prostate cancer cell survival and proliferation, major NF-κB signaling pathways in prostate cancer, and the crosstalk between NF-κB and androgen receptor signaling. Several NF-κB-induced risk factors in prostate cancer and their potential for therapeutic targeting in the clinic are described. A better understanding of the inflammatory mechanisms that control the development of prostate cancer and resistance to androgen-deprivation therapy will eventually lead to novel treatment options for patients.

## 1. Introduction

### 1.1. The NF-κB Family of Transcription Factors

Inflammation and cancer have been described in the medical literature for millennia [1], and there have been suggestions of a link between inflammation and cancer for centuries [2], but the significant overlap in molecular mechanisms between inflammation and cancer has only begun to be appreciated in recent decades [3,4]. Major players in both inflammation and cancer are the NF-κB transcription factors, which thus need to be tightly regulated [5]. The link between NF-κB and cancer was already apparent at its early discovery, since the oncogenic viral NF-κB family protein v-Rel was identified as a κB DNA binding transcription factor [6]. Many more viral proteins that induce inflammatory pathways also promote cancer development [7]. In addition, many mutations in NF-κB signaling proteins that cause dysregulated pro-inflammatory signaling also induce cancer [8]. 

**Figure 1:**
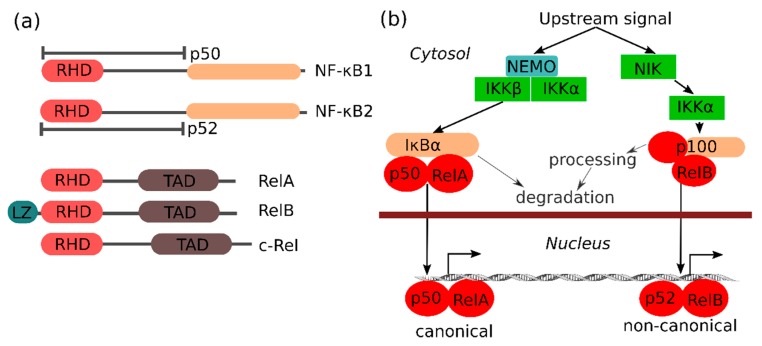
Structure and signaling of NF-κB and NF-κB-associated components. (**a**) Domain composition of class I and II NF-κB family members. Rel homology domain (RHD), transactivating domain (TAD), leucine zipper (LZ), IκB ankyrin repeat domain is colored in orange (**b**) A simple overview of the canonical and non-canonical NF-κB signaling pathways. Kinases are presented in green, NF-κB subunits in red, and IκB proteins in orange. In both pathways, phosphorylation leads to the proteasomal removal of inhibitory domains (e.g., in case of p100) or proteins (in case of IκBα) that prevent the NF-κB dimers from entering the nucleus to activate transcription. Alternative canonical NF-κB activation pathways via IKKε/TBK1 or IKK-independent pathways are not shown.

The NF-κB family of transcription factors consists of different proteins (NF-κB1 (also known as p105), NF-κB2 (also known as p100), RelA (also known as p65), RelB and c-Rel) that are defined by their Rel homology domain (RHD), which is important for protein–protein interactions and assembly of an active transcription factor complex (Figure 1a). The transactivating (class II) NF-κB family members (RelA, RelB and c-Rel) are kept inactive by binding to the ankyrin repeat domain of inhibitor of NF-κB (IκB) family members, including p100, p105 and IκB proteins, which prevent NF-κB from entering the nucleus. Notable for p100 and p105 is that they can be processed into the p52 and p50 subunits, respectively, removing their ‘inhibitory’ ankyrin repeat domain. The p50 and p52 NF-κB (class I) subunits are DNA binding, but do not have trans-activation activity by themselves. NF-κB binds to specific DNA sequences as multiple homo- or heterodimers, with only dimers containing a class II subunit with a transactivation domain (TAD) being transcriptionally active. RelA and c-Rel can be active on their own as homodimers, whereas RelB depends on heterocomplexes with p50 or p52. The different NF-κB complexes are not redundant, and specific NF-κB complexes are activated via two major pathways known as the canonical and the non-canonical NF-κB pathway, respectively (Figure 1b). The standard canonical pathway for NF-κB activation relies on the canonical IKK (IκB kinase) complex consisting of IKKα, IKKβ and IKKγ (NEMO), and is best described for several pro-inflammatory stimuli such as tumor necrosis factor (TNF). Upon stimulation of cells, IKKβ phosphorylates IκBα, which leads to its subsequent ubiquitination and proteasomal degradation, thus releasing and allowing the p50/RelA complex to enter the nucleus. In certain conditions (e.g., downstream of certain viral receptors or in certain cancers), the IKKβ-related kinases IKKε and TBK1 can also activate NF-κB via IκBα, RelA and c-Rel phosphorylation [9], while IKK-independent mechanisms have also been reported (e.g., in response to DNA damage or hypoxia). In contrast to the critical role of IκBα in the canonical NF-κB pathway, the other IκB family members (IκBβ, IκBε, IκBζ, Bcl-3, IκB_NS_) seem to play more subtle roles (see below). The non-canonical NF-κB pathway, which is best known for its role in lymphoid organ development, is independent of IKKβ, but is regulated by the kinases NIK and IKKα. Notable for the non-canonical pathway is that it induces processing of the p100 pre-protein into the active p52 subunit. In the non-canonical pathway, p52 primarily activates NF-κB transcription, together with the transactivating RelB subunit (Figure 1b). The IKKα-related kinase IKKε can, however, also promote p52 transactivation by RelA [10]. 

The c-Rel NF-κB subunit is strongly associated with the development of many types of cancer [11]. In contrast to p50/RelA and p52/RelB, the regulation of c-Rel is less clear, but transcriptionally active complexes can occur as homodimers or as c-Rel/p50, c-Rel/RelA and c-Rel/p52 heterodimers [12]. In some cellular systems, RelB can act as an inhibitor of c-Rel-specific transcriptional responses [13], and c-Rel has also been suggested to be specifically negatively regulated by IκBε [14]. In contrast, IκBβ seems to promote NF-κB-dependent responses [15], especially c-Rel-dependent and RelA homodimer transcriptional activity [16,17].

NF-κB subunits do not exclusively interact with each other, and other “hybrid” active transcription complexes can also occur where DNA binding is provided by p50 or p52, but with transactivation by a different kind of protein. For example, the IκB family members Bcl-3 and IκBζ have transactivating activity and can form nuclear heterocomplexes with p52 or p50 to promote specific NF-κB-driven transcription [18]. Bcl-3 in complex with NF-κB is known to promote pro-survival and pro-proliferation transcription and suppress pro-inflammatory transcription after protein kinase C (PKC) activation by phorbol ester stimulation [19,20], which is important for the development of certain cancers.

### 1.2. Prostate Cancer: Role of Inflammation and Androgen Hormones

The prostate is a slightly bigger than walnut-sized gland (~20 g) in men, which produces the alkaline part of the seminal fluid to protect sperm against the acidic pH environment in the vagina. Prostate cancer is the most commonly occurring form of cancer in men, with 40–70% of men between 60 and 70 years old in Western countries dying from other causes have prostate cancer identified during autopsy, and the frequencies are even higher at more advanced ages [21]. A smaller (~5 g) corresponding organ, the female prostate (aka Skene’s gland), is actually also present in females of various species, but its function is unclear, and the low awareness of this organ in women might lead to misdiagnosis of diseases associated with it [22,23]. Cancer originating from the female prostate appears very similar to male prostate cancer, but it is most likely extremely rare [24]. The only non-human animal known to also spontaneously develop prostate cancer is dogs, where the disease is very similar to that of humans, except that it is rarer and more aggressive [25]. The similarities between dog and human prostate cancer make dogs good animal models for the disease [26]. 

The prevalence of prostate cancer in men might be higher in Western countries due to being a comorbidity to obesity-related diseases like insulin resistance and constant inflammation [27,28,29,30]. Inflammatory signals in the prostate will enhance proliferation of otherwise normal epithelial cells [31], which could be a starting point for cancer development. Further indications for an important role of inflammation in prostate cancer initiation is provided by genetic analyses that have found associations between inflammation-related genes and prostate cancer risk [32]. Inflammatory signals and insulin-like signaling are, however, also involved in normal prostate growth and development [33]. Despite prostate cancer typically being a slowly developing and benign form of cancer [34], the high frequency of cases in the male population still results in a significant number of malign cases, making prostate cancer the 2nd leading cause of death by cancer in males in the US [21,35]. Malignant prostate cancer cells migrate to lymph nodes and can end up in bone, brain, liver and lungs, and liver metastasis, in particular, seems associated with poor survival [36,37]. 

Just as breast cancers are usually addicted to the female sex hormone estrogen, initially, prostate cancer typically also starts addicted to the male sex hormone androgen [38,39]. One treatment of prostate cancer has thus been to limit the amount of androgen, which could be achieved by castration [40], but tumor cells can start to produce their own androgens and become castration-resistant [41]. Prostate cancer in dogs is typically androgen-independent and aggressive, but castration prior to development of prostate cancer is still protective, indicating a role for androgens in the early stages also in dog prostate cancer [25]. Castration induces cell death and inflammation in the prostate, which needs to be controlled by macrophages [42], but these immunosuppressive myeloid cells can also contribute to the development of castration-resistant prostate cancer [43]. Noteworthy, induction of endogenous androgen biosynthesis in prostate cancer cells is at least partially driven by NF-κB, and might thus also be promoted by inflammation induced by castration [44]. 

The rare cases of female prostate cancer, together with reports of the development of androgen-independent aggressive forms of prostate cancer in transgender women up to 30–40 years after male-to-female corrective surgery (including castration), challenge the concept of androgens being required in the initial phases of prostate cancer [24,45,46]. In contrast, animal experiments with androgen injections in females have revealed growth of the female prostate, coupled with elevated secretory activity and serum prostate-specific antigen (PSA) levels [47]. These animal experiments also seem to reflect what happens in humans, since elevated PSA levels have also been observed in transgender men (female-to-male) undergoing androgen treatment [48]. There are, however, currently no reports of elevated female prostate cancer risk in transgender men after androgen treatments compared to cisgender (i.e., sex at birth is the same as the identified gender [49]) women. The rare cases of typically sex hormone-dependent cancer types in the “wrong” sex (e.g., cis-female prostate cancer and cis-male breast cancer) could perhaps also provide further insights on sex hormone-independent or alternative variations of sex hormone dependency in the early development of these cancer types. For example, the androgen receptor (AR) and the estrogen receptor (ER) β seem to promote male breast cancer, whereas both sex hormone receptors seem to inhibit female breast cancer [39].

Androgen signal deprivation is nowadays typically achieved by AR antagonists that might also be efficient against some cases of castration-resistant prostate cancer that depend on alternative androgen metabolism [50]. A remaining problem, however, is that prostate cancer also evolves into an AR-independent cancer after long-term treatment with AR antagonists, and AR antagonist-resistant cells may show elevated metastatic growth [51,52]. Identification of the mechanisms responsible for the development of androgen deprivation resistance is thus an important challenge in devising durable prostate cancer treatment options. Potentially important pieces of the puzzle for both prostate cancer androgen deprivation resistance and prostate cancer initiation are pro-inflammatory signals, which can be prostate cancer cell intrinsic, come from associated immune cells, or come from an interaction between the two [53,54,55,56,57,58]. Because of the many links between inflammatory signals and the development and malignancy of prostate cancer, targeting NF-κB has been suggested as a promising therapeutic option [59].

## 2. NF-κB in Prostate Cancer

### 2.1. NF-κB in Prostate Cancer Cell Survival and Proliferation

Several studies have demonstrated that NF-κB promotes cell survival, proliferation and invasion in prostate cancer [60,61,62]. Of the different NF-κB family subunits, especially p52 [63] has been shown to be important, but the transactivating subunits RelA [64], RelB [65,66] and c-Rel [67] have all also been implicated in prostate cancer. Notably, Bcl-3 is also important for prostate cancer cell survival during chemotherapy [68]. The development of treatment resistance is thought to depend on so-called cancer stem cells [69], and an AR-negative prostate cancer stem cell population with constitutive NF-κB activity was recently discovered [70]. It is possible that the AR-negative prostate cancer cells grow out of this stem cell population during androgen deprivation therapy, which means that NF-κB inhibition could be a promising target to prevent the evolution of AR-negative prostate cancer cells. NF-κB plays a key role in the development of castration or AR antagonist resistance, and combination treatments with NF-κB and AR inhibitors have shown promising results [71,72]. The role of NF-κB in development of castration-resistant prostate cancer has also been verified in mouse genetic models with constant NF-κB activation in prostate cancer cells [73]. A further indication for the role of NF-κB in prostate cancer proliferation is the observation that the NF-κB-inhibiting drug Aspirin and its active metabolite salicylic acid (SA) are both capable of significantly inhibiting growth of the androgen-independent prostate cancer cell line DU-145 [74]. Aspirin could, however, also act on prostate cancer via cyclooxygenase inhibition (COX) [75], which drives prostaglandin biosynthesis, and prostaglandins are important for prostate cancer cell growth [76,77,78,79]. Since free SA also shows inhibitory effects, and free SA cannot inhibit COX, the most likely explanation is, however, a direct effect of SA on NF-κB signaling at many different levels [80]. Interestingly, SA has also been shown to directly bind high mobility group box protein 1 (HMGB1), whose overexpression in prostate cancer is closely associated with the proliferation and aggressiveness of tumor cells, and which is known to promote the epithelial-to-mesenchymal transition in prostate cancer PC3 cells via the receptor for advanced glycation end products (RAGE)/NF-κB signaling pathway [81,82]. Epidemiological studies also indicate that long-term use of Aspirin or other non-steroidal anti-inflammatory drugs are protective against prostate cancer [83].

### 2.2. Crosstalk between NF-κB and Androgen Receptor Signaling

**Figure 2:**
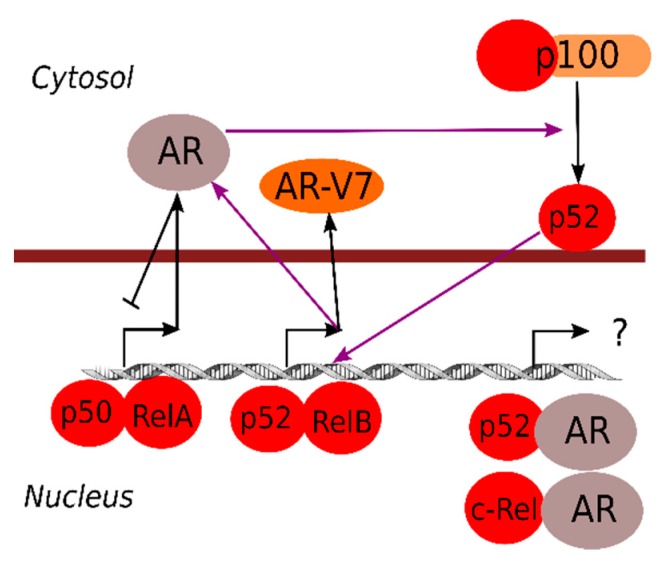
Schematic overview of crosstalk between AR and various NF-κB family members. Both p50/RelA and p52/RelB can drive AR expression, and p52/RelB can also drive expression of an androgen-independent AR splice variant (AR-V7). AR stimulation, in turn, represses canonical (p50/RelA) but promotes non-canonical (p52/RelB) NF-κB signaling. A potential feed-forward loop between AR and non-canonical NF-κB signaling is highlighted with purple arrows. AR can also act as a transactivator in complex with NF-κB family members, but the significance of the transcripts downstream of these hybrid transcription factors is currently unknown.

Just like the estrogen receptor and other steroid receptors are well-known inhibitors of NF-κB responses [84,85], the AR can also repress NF-κB-dependent transcription (Figure 2) [86,87]. In line with this, decreasing androgen levels or blocked AR signals are associated with elevated inflammatory markers [88,89]. 

AR specifically blocks canonical NF-κB (RelA/p50)-driven expression, but seems to positively induce non-canonical NF-κB activation (processing to mature p52) [90]. Activation of non-canonical NF-κB could thus be a very important step in the development of androgen independence, since loss of androgen repression of NF-κB target genes is associated with poor prognosis in metastatic prostate cancer [91]. The p52 subunit can also activate AR signaling, which, in addition to induction of metabolic reprogramming of prostate cancer cells through induction of genes for glucose uptake and metabolism, contributes to androgen-independent growth [63,92]. One mechanism for p52-induced androgen-independent AR signaling is the induced expression of a constitutively active AR splice form (AR-V7) [93,94]. On the other hand, the canonical IKKα and IKKβ upstream of NF-κB might also directly influence AR activity by phosphorylation [95], which might represent another level of cross-talk between NF-κB and AR. 

Specific NF-κB subunits can also form heterodimers with AR. For example, an AR/p52 complex has been shown to be important for prostate cancer growth [96], an AR/c-Rel complex whose functional role is still unknown [11,97]. Also, high nuclear RelA, together with high nuclear AR expression, is associated with poor prostate cancer outcome, indicating the existence of other relevant NF-κB/AR interactions [98,99]. Combination treatments with pro-inflammatory stimuli and androgen has also revealed unique responses compared to each stimulation alone [100], indicating that NF-κB/AR complexes have specific functions and can induce specific transcriptional programs. 

## 3. NF-κB Signaling Pathways in Prostate Cancers

### 3.1. Innate Immune Receptors in Prostate Cancer

Several innate immune receptors can be activated in the prostate as a consequence of infection or other stresses to cause prostate inflammation (prostatitis). In prostate cancer, the same receptors can also promote or worsen prostate cancer progression. The intracellular inflammasomes NLRP3, NLRP12 and AIM2 can be triggered by several stimuli, and are responsible for IL-1 and IL-18 expression in prostate cancer [101,102]. The lectin-like LOX-1 receptor, which detects oxidized low-density lipoprotein and advanced glycation endproducts (AGE), signals to NF-κB in prostate cancer and could represent another mechanism for comorbidities between metabolic syndrome and prostate cancer [103]. Another AGE receptor, RAGE, detects the danger signal/pro-inflammatory cytokine HMGB1, which plays an important role in prostate cancer metastasis development via NF-κB signaling [81]. The role of Toll-like receptors (TLRs), a major class of innate immune receptors, is a more complicated image. Different TLRs can either promote or inhibit prostate cancer growth [104]. Loss of the critical downstream TLR signaling component MyD88 significantly worsens the phenotypes in mouse models of prostate cancer, but these effects appear to be indirect and reflect the role of MyD88 in immune cells [105]. On the other hand, responses to viral signals via TLR3 or RIG-I, especially, seem to be highly detrimental to prostate cancer cells [106]. In line with this, prostate cancer cells can be very sensitive to interferons [107].

### 3.2. PKC and PKC-Related Signals in Prostate Cancer

**Figure 3:**
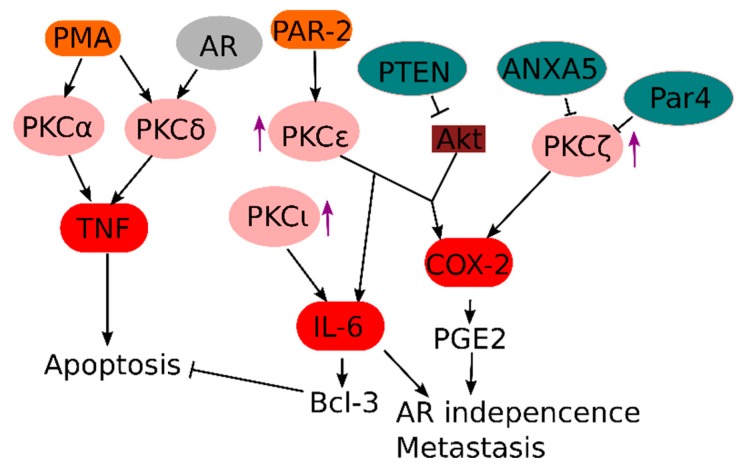
Overview of the role of PKC-dependent signaling in prostate cancer. PMA-induced activation of both PKCα and PKCδ has been associated with prostate cancer cell apoptosis due to autocrine TNF production, whereas elevated expression (arrow pointing up) of the other PKCs is associated with survival and aggressiveness of prostate cancer via multiple other mechanisms (see text for details). At least one of the pro-oncogenic PKCs (PKCε) can also be activated by upstream signaling.

One major class of oncogenic pro-inflammatory signaling proteins are members of the PKC family, which were discovered as the kinases responsible for the oncogenic effects of phorbol esters (i.e., PMA) [108]. Due to their common role in inflammation and cancer, PKCs are generally considered attractive therapeutic targets. For example, the broad range PKC inhibitor sotrastaurin has been used in clinical and pre-clinical trials, both as immunosuppressant [109,110] and cancer treatment [111]. Several PKCs have been implicated in prostate cancer with different downstream effects (Figure 3). 

In prostate cancer, PKC activity is induced after AR inhibition, which could contribute to NF-κB-driven AR independence [112]. PKC-associated NF-κB responses in prostate cancer are specifically dependent on c-Rel, which induces transcripts associated with angiogenesis, inflammatory responses and cell motility [67]. PKCε is one upstream signaling activator of NF-κB in prostate cancer cells, which is often overexpressed in metastatic prostate cancer, and many studies argue for a causal link between PKCε overexpression and prostate cancer development [60,61,113,114]. Interestingly, it has been shown that the trypsin-activated proteinase-activated receptor (PAR)-2 signals via PKCε to induce cell proliferation, which could explain the pro-inflammatory effect of various secreted proteases from prostate cancer cells [115]. Recently, it was also discovered that PKCε acts synergistically with loss of *PTEN* (which results in constitutive Akt activation) for NF-κB activation, leading to elevated expression of the pro-inflammatory prostaglandin biosynthesis enzyme COX-2 [116]. 

In contrast, activation of other PKC isoforms, such as PKCα and PKCδ, by PMA stimulation of prostate cancer cells rather induces cell death due to autocrine TNF and TRAIL signaling [117]. This inflammation-induced autocrine suicide could theoretically be exploited as a therapy by blocking the pro-survival pathways downstream of TNF [118]. Interestingly, PKCδ expression in prostate cells is dependent on androgen signals, suggesting that therapeutic targeting of PKCδ might be highly dependent on the level of AR signaling in the prostate cancer. 

Surprisingly, atypical PKCs, which require neither Ca^2+^ nor diacylglycerol for activation, seem to also play a role in NF-κB activation in the context of prostate cancer [119]. Expression of the atypical PKCλ or PKCι promotes hormone-independent growth of prostate cancer cells through the NF-κB dependent induction of pro-inflammatory proteins like IL-6, and genetic variants of PKCι have been associated with elevated prostate cancer risk [120,121]. IL-6, in turn, can induce expression of the anti-apoptotic Bcl-3 protein via STAT3 signaling, which can contribute to prostate cancer cell survival [20]. Knock-out of the prostate apoptosis response 4 (*Par4*), a pro-apoptotic tumor suppressor gene, results in spontaneous development of prostate cancer in mice, possibly due to loss of negative regulation of the atypical PKCζ and elevated expression of the anti-apoptotic protein XIAP [122]. Also, inhibition of PKCζ expression by Annexin A5 seems important in repressing COX-2 expression in prostate cancer cells [123]. This is in line with several observations showing that elevated expression and alternative splicing of PKCζ promotes an aggressive prostate cancer phenotype [124,125]. Of interest, the anti-rheumatic drug and atypical PKC inhibitor aurothiomalate is highly efficient against prostate cancer cells [126]. 

More distantly related members of the PKC superfamily like PKN1, PKN2 and PRKD3 (PKCυ) were shown to play an important role in prostate cancer motility [127,128], and inhibition of PKN1 has been shown to be an attractive therapeutic strategy [128,129]. On the other hand, another PKC superfamily member, PRKD1 (PKCµ), is inversely associated with prostate cancer malignancy [130], illustrating the complex role of the PKC superfamily in prostate cancer.

### 3.3. GPCR Signaling in Prostate Cancer

Several G protein-coupled receptors (GPCRs) are associated with poor prognosis in prostate cancer and might represent interesting pharmacological targets [131,132,133,134,135,136,137,138,139]. Many GPCRs that indicate poor prognosis of prostate cancer signal via the Gα_12_/Gα_13_—RhoA axis via PKC for NF-κB activation [140,141,142], but GPCRs also induce several other transcriptional regulators relevant for cancer, like AP-1, MRTF-A and YAP [140]. In agreement with this, parallel blocking of both the Akt and the NF-κB pathway seems to be important in eliminating the effect of hyperactive GPCR signaling in prostate cancer cells [143]. The most notable GPCR for prostate cancer is the thrombin receptor PAR-1, which is known to signal to NF-κB [144], and is also a well-known poor prognostic marker in prostate cancer [145,146,147,148]. Prostate cancer cells produce thrombotic extracellular vesicles, which can in turn activate the thrombin receptors on prostate cancer cells or surrounding stromal cells [149,150]. Low-dose thrombin inhibition has been suggested as a potential prostate cancer treatment [151]. Also, the anti-inflammatory drug Aspirin, which has been shown to have protective effects against prostate cancer [83], could in principle partially affect prostate cancer cells through its anti-thrombotic activity. The association of GPCR signaling with poor prognosis in prostate cancer is a common theme shared with other solid tumors like breast cancer [152]. Apart from dysregulation at the receptor level, activating mutations or overexpression of the Gα_12_, Gα_13_ and RhoA downstream signaling components were shown to be risk factors in prostate cancer [153,154,155]. Furthermore, RhoA has been shown to activate PKCζ, which in turn leads to enhanced cell proliferation [156]. Overexpression of other GPCR downstream signaling components like the guanine nucleotide exchange factor Vav3 in the prostate epithelium was also shown to be sufficient for induction of prostatitis and NF-κB-dependent prostate cancer development [157].

## 4. NF-κB-Induced Risk Factors in Prostate Cancer

The different NF-κB complexes induce a wide range of transcriptional responses, which in turn can contribute to prostate cancer malignancy. Targeting downstream effects could be very attractive as an alternative to broad inhibition of signaling pathways. In cancers, however, the concept of “upstream” and “downstream” is not always clear. For example, IKKε is a downstream target that is induced by NF-κB and inhibited by AR in prostate cancer cells, and AR-negative prostate cancer cells show constitutive IKKε activation [158]. IKKε, in turn, can induce an NF-κB-independent expression of IL-6, which contributes to malignancy and androgen independence [159]. IKKε could theoretically also contribute to prostate cancer malignancy by phosphorylating and inactivating tumor suppressors like CYLD [160,161]. Supporting the importance of IKKε, specific inhibitors have been shown to be potentially interesting therapeutic options in prostate cancer [162]. 

Apart from the well-described contribution of IL-6-induced responses to AR antagonist resistance [163], IL-1β can also contribute to the development of resistance by inducing a gene expression profile in androgen-dependent cells that resembles that of androgen-independent cells [164]. In addition to the role of inflammasomes in IL-1β production, elimination of PKCε in prostate cancer cells also blocks production of IL-1β [61]. IL-1β expression is high in AR-negative prostate cancer cells, but IL-1β also represses AR expression and promotes bone metastasis [165,166,167,168,169]. Interestingly, the IL-1 inhibitor Anakinra is able to suppress prostate cancer bone metastasis and could be an interesting therapeutic option [165]. Also, expression of RANK and its ligand by prostate cancer cells promotes epithelial-to-mesenchymal transition and bone metastasis [170,171], indicating a potential for RANK targeting in prostate cancer. In addition to IL-1β and IL-6, several other cytokines have been associated with prostate cancer. IL-30 (IL-27 p28 form) expression from prostate cancer cells is associated with advanced-grade prostate cancer and IL-30 stimulates prostate cancer cell proliferation in vitro [172]. IL-8 is expressed specifically in AR-independent cells, and expression of IL-8 in AR-dependent cells also induces AR independence [173]. IL-8 expression also confers resistance to cytotoxic chemotherapy in prostate cancer cells [174]. This pro-survival role is shared with IL-12, whose depletion by antibodies causes prostate cancer cell death via IFNγ in vivo [175]. Recently, also IL-23 produced by myeloid-derived suppressor cells was shown to activate the androgen receptor pathway in prostate tumor cells, promoting cell survival and proliferation in androgen deprived conditions [43]. Most interestingly, IL-23 blocking antibodies could restore sensitivity to androgen-deprivation therapy in mice.

Although TNF has been associated with cell death in stimulated prostate cancer cells in vitro [117,176], an in vivo model using TNF receptor 1 knock-out mice suggests, rather, that TNF promotes prostate cancer proliferation [177]. High prostatic expression of TNF has also been associated with poor clinical outcome [178]. To exploit this therapeutically, some pro-inflammatory cytokines such as TNF could perhaps be made to kill cytokine-producing prostate cancer cells, for example by combination treatments with IFNγ [107,179,180]. Not all pro-inflammatory cytokines are, however, beneficial for prostate cancer cell growth, survival and metastasis. IL-18 is produced in prostate cancer cells after IFNα treatment, and high IL-18 expression is associated with beneficial clinical effects [181,182]. In line with this and in contrast to IL-1β, overexpression of IL-18 in prostate cancer cells inhibits tumor growth in vivo [183]. Another indication for an important role of IL-18 in prostate cancer is that some genetic variants of the IL-18 promoter are associated with elevated prostate cancer risk in different populations [184,185,186]. Also, the pro-inflammatory cytokine IL-33 is preferentially lost in metastatic prostate cancer in order to avoid immune cell triggering [187]. Apart from the direct pro-inflammatory signals, chemokines can also be induced in prostate cancer cells or other cells by inflammatory cytokines, which in turn can promote migration of prostate cancer cells and bone metastasis [188,189,190]. 

The interaction between prostate cells and the immune microenvironment can be far more complex. Not only can the pro-inflammatory signals associated with activated (“M1”) macrophages contribute to prostate cancer through enhanced proliferation and aggressiveness, anti-inflammatory cytokines (like IL-4) associated with alternatively activated (“M2”) macrophages can also contribute to pathology by activating AR signaling [191]. Also, expression of IL-4 in androgen-dependent prostate cancer cells can induce androgen-independent growth [192]. In addition, IL-4 promotes the clonogenic expansion of prostate cancer stem-like cells [193]. On the other hand, genetic variants characterized by low production of IL-10 are associated with higher risk of prostate cancer recurrence [194], indicating that the anti-inflammatory role is dominant. It should be noted that many studies showing a role for specific cytokines are based on overexpression and await confirmation at more physiological expression levels. Also, many NF-κB activating signals and downstream factors can probably cause similar aggressive and AR-independent prostate cancer phenotypes, implying that it will be important to find those cytokines or mediators that play a non-redundant function in the promotion of aggressive prostate cancer in vivo. 

## 5. Conclusions and Perspectives

Androgen deprivation therapy has become the main prostate cancer therapy for patients at different stages of disease. However, a considerable fraction of patients receiving such treatments ultimately progress to a more aggressive disease, developing androgen-independence. Acquiring a better understanding of the mechanisms that control the development of prostate cancer and resistance to androgen-deprivation therapy remains an unmet clinical need. There is significant positive and negative crosstalk between steroid hormone receptor signaling (including androgen receptors) and inflammatory signaling mediated by NF-κB and other transcription factors. In the context of therapeutic targeting, it is, therefore, highly interesting to identify central inflammatory signaling nodes that are responsible for the development of androgen independence. Up until recently, we have been limited to druggable targets in the signaling pathways as therapeutic options. Advances in genome editing (with for example CRISPR/Cas9) coupled with efficient and reliable targeting to prostate cancer cells will however open up for a wider selection of potential targets to manipulate therapeutically [195]. Also, several cytokines that have been implicated in prostate cancer (e.g., IL-1β, IL-6, IL-12, IL-23) and for which cytokine blocking antibodies are already in the clinic or under clinical evaluation for the treatment of autoimmune diseases, deserve to be clinically evaluated in men that have lethal prostate cancer. However, more general anti-inflammatory drugs could also be beneficial, in combination with other types of treatment. One such general anti-inflammatory drug that seems to stand out is Aspirin, which not only affects inflammatory signaling leading to NF-κB activation at several levels [80], but also shows additional therapeutic activities (like anti-thrombotic activity) and directly targets molecules like HMGB1 [82] and COX-2 [75], which have been shown to be important in prostate cancer. As with any disease mechanism studied mainly in mouse models, the prevalence and importance of certain risk factors in humans remains to be determined, in most cases. Patient stratification and predictive biomarkers will probably be crucial to successfully implement novel treatments in the clinic.

Noteworthy, prostate and breast cancers are in many ways similar [39], not only in that they both generally start off as sex hormone-dependent, but they also share mechanisms of inflammation-induced development of hormone independence. There is a striking overlap in pro-inflammatory signaling molecules leading to NF-κB activation and contributing to malignancy in both breast and prostate cancer (e.g., PKCε, IKKε and GPCRs). It might therefore be interesting to further explore these common themes on inflammation and sex hormone independence.
